# Therapeutic potential of the chemical composition of *Dendrobium nobile* Lindl.

**DOI:** 10.3389/fphar.2023.1163830

**Published:** 2023-07-11

**Authors:** Chenxi Fan, Xin Sun, Xin Wang, Hongsong Yu

**Affiliations:** ^1^ Department of Immunology, Special Key Laboratory of Ocular Diseases of Guizhou Province, Zunyi Medical University, Zunyi, Guizhou, China; ^2^ Special Key Laboratory of Gene Detection and Therapy of Guizhou Province, School of Basic Medical Sciences, Zunyi Medical University, Zunyi, Guizhou, China

**Keywords:** chemical composition, *Dendrobium nobile* Lindl., application, diseases, treatment

## Abstract

*Dendrobium nobile* Lindl. belongs to the genus *Dendrobium* of the orchid family and is a valuable herbal medicinal material. The information in this paper has been collected from the scientific literature databases including PubMed, Google Scholar, Web of Science, SciFinder, China National Knowledge Infrastructure, published books, Ph.D., and M.S. dissertations systematically in recent 20 years. “*Dendrobium nobile* Lindl.,” “chemical composition,” “pharmacological activities,” and “diseases” were used as search terms to screen the literature. The collected chemical compositions are classified and summarized according to their different chemical structures, and the clinical disease treatment effects of *Dendrobium nobile* Lindl. are classified and summarized based on their pharmacological activities and different experimental disease models. Recent studies have revealed that *Dendrobium nobile* Lindl. contains chemical components such as alkaloids, bibenzyls, sesquiterpenes, phenanthrenes, and polysaccharides, and that its pharmacological activities are closely related to the chemical components, with pharmacological activities such as anti-tumor, anti-aging, immune enhancement, hypoglycemic, and anti-cataract. Currently, researchers are conducting extensive and detailed studies on *Dendrobium nobile* Lindl. and research experiments on its chemical constituents in the treatment of various clinical diseases. Therefore, the purpose of this paper is to review the chemical composition of *Dendrobium nobile* Lindl. and its experimental studies in the treatment of diseases and to provide a scientific reference for the future application of *Dendrobium nobile* Lindl. in the treatment of diseases.

## 1 Introduction

*Dendrobium nobile* Lindl. is a valuable Chinese medicinal material belonging to the genus *Dendrobium* of the orchid family (Lam et al., 2015). It is the earliest identified species of *Dendrobium* in ancient Chinese medicinal herbs and one of the main varieties of medicinal dendrobiums in China. It has a long history of application and is one of the most valuable medicinal plants in Guizhou province, China. Due to its excellent medicinal properties, *Dendrobium nobile* Lindl. was listed as the original species of medicinal dendrobium in the 2010 edition of the Chinese Pharmacopoeia, attracting the attention of many researchers. The related literature shows that *Dendrobium nobile* Lindl. is a medicinal plant containing mainly alkaloids, bibenzyls, sesquiterpenes, phenanthrenes, polysaccharides, and other chemical compositions. Its pharmacological effects appear mainly as anti-tumor, anti-aging, immune enhancing, hypoglycemic, and anti-cataract (Huang et al., 2017; Chao et al., 2018). Recently, the active constituents of *Dendrobium nobile* Lindl., especially alkaloids and polysaccharides, have been found to have significant therapeutic effects against tumors, hypoglycemia, and nervous system diseases, and some proprietary herbal medicines based on *Dendrobium nobile* Lindl. has been used in the treatment of a number of diseases. With the continuous development of modern molecular chemistry and pharmacology, various chemical constituents have been isolated from *Dendrobium nobile* Lindl., and their multiple pharmacological actions have been revealed. In addition, chemical constituents have been studied for the treatment of various diseases. Therefore, the purpose of this paper is to summarize the experimental studies on the chemical constituents of *Dendrobium nobile* Lindl. and to provide a scientific reference for future applications of *Dendrobium nobile* Lindl. in the treatment of diseases.

## 2 Chemical composition of *Dendrobium nobile* Lindl.

In recent years, the chemical constituents of *Dendrobium nobile* Lindl. have been systematically studied, revealing that it contains a wide variety of chemical constituents. Based on its structure, the main chemical constituents of *Dendrobium nobile* Lindl. can be divided into alkaloids, polysaccharides, phenanthrenes, sesquiterpenes, and bibenzyls (Table 1), among which alkaloids are the characteristic composition of *Dendrobium nobile* Lindl.

**TABLE 1:** Chemical composition of *Dendrobium nobile* Lindl.

Chemical composition of *Dendrobium nobile* Lindl.	Part of the *Dendrobium nobile* Lindl. used to extract	Reference
Alkaloids	Leaves and stems	Zhou et al. (2017)
		Shi et al. (2017)
		Wang et al. (2016b)
Polysaccharides	Stems, leaves, flowers, and roots	Jiang and Luo (2011)
		Yang et al. (2010)
Bibenzyls	Stems	Xiao et al. (2016)
		Zhang et al. (2008a)
Phenanthrenes	Stems	Zhou et al. (2018b)
		Ling et al. (2021)
Sesquiterpenes	Stems	Wang X. Y. et al. (2019)
Tannins	Stems	Hen et al. (2013)
Fluorenones	Stems	Zhou et al. (2018a)
Coumarins	Stems	Zhou et al. (2018b)
Lignans	Stems and roots	Zhang et al. (2008a)

### 2.1 Alkaloids

**FIGURE 1:**
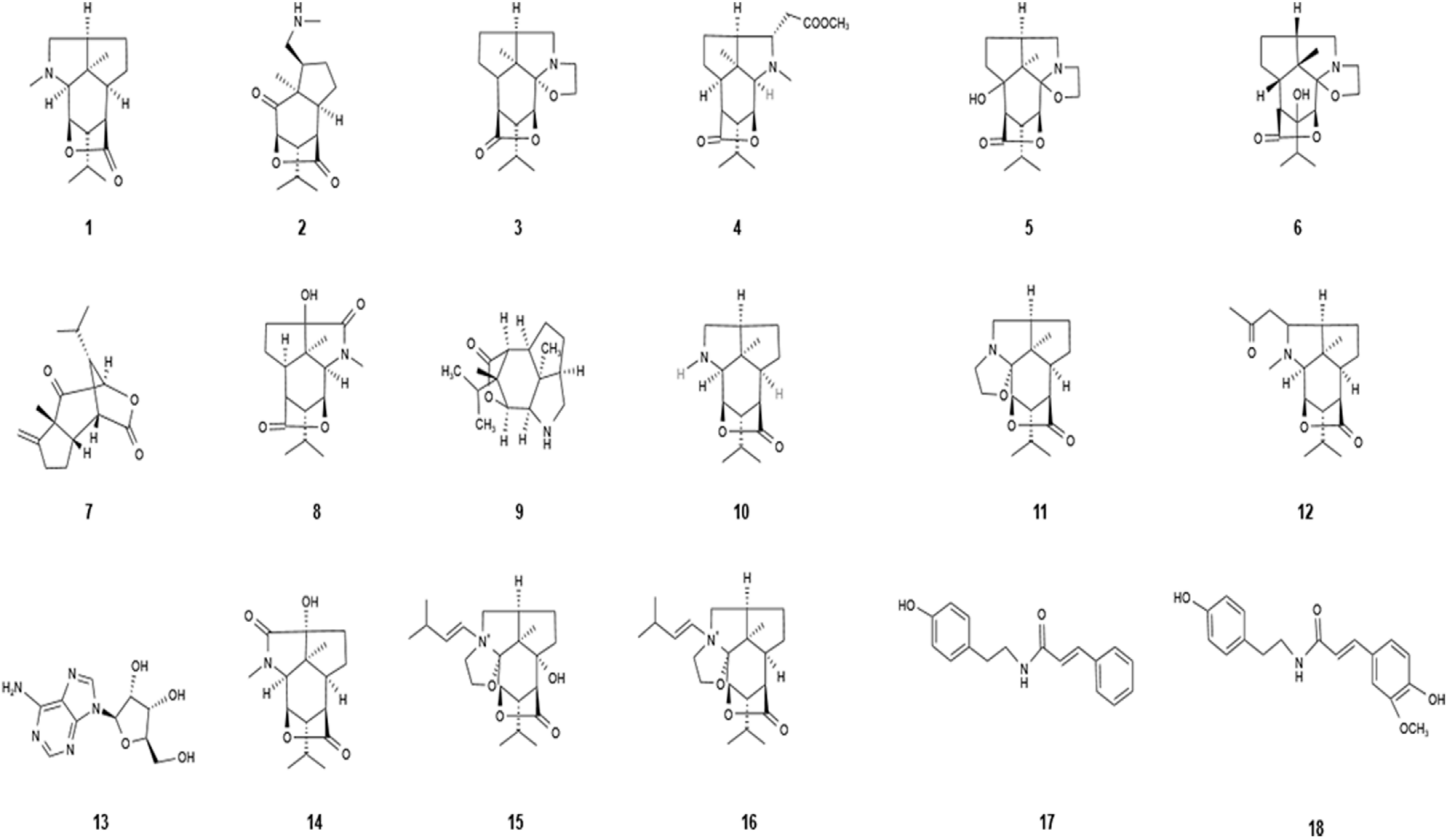
Diagram of the chemical structure of alkaloids isolated from *Dendrobium nobile* Lindl. Different numbers represent the different compounds: 1, dendrobine; 2, nobilonine; 3, dendroxine; 4, dendrine; 5, 6-hydroxydendroxine; 6, 8-hydroxydendroxine; 7, nobilomethylene; 8, 3-hydroxy-2-oxodendrobine; 9, nordendrobine; 10, mubironine B; 11, dendroxine; 12, dendronobiline A; 13, adenosine; 14, 9-hydroxy-10-oxodendrobine; 15, N-isopentenyl-6-hydroxydendroxinium; 16, N-isopentenyldendro xinuum; 17, N-trans-cinnamoyltyramine; and 18, N-trans-feruloyltyramine.

Alkaloids are natural organic compounds containing nitrogen heterocycles found in plants, and these nitrogen atoms determine the alkalinity of alkaloid compounds (Heinrich et al., 2021). Alkaloids are the characteristic constituents of *Dendrobium nobile* Lindl. and are the main active substances. The alkaloids were first isolated and purified from the stems and leaves of *Dendrobium nobile* Lindl. in 1932, and it was found that alkaloids were mainly distributed in the stems of *Dendrobium nobile* Lindl., which led researchers to generally adopt the stem part of *Dendrobium nobile* Lindl. for the extraction and isolation of alkaloids in the subsequent process (Zhou et al., 2017; Shi et al., 2017; Wang et al., 2016b). The alkaloids of *Dendrobium nobile* Lindl. have anti-tumor, hypertension-reducing, and nervous system-protecting effects (Mou et al., 2021). In recent years, several research studies have been conducted on the alkaloids of *Dendrobium nobile* Lindl., and various types of alkaloids are constantly being discovered. To date, the structural and molecular formula of various alkaloids have been identified, including dendrobine, nobilonine, dendroxine, dendrine, 6-hydroxydendroxine, and 8-hydroxydendroxine (Chen and Shi, 2016). The alkaloids isolated from *Dendrobium nobile* Lindl. are listed in Table 2, and their chemical structures are shown in Figure 1. The reason why alkaloids are the unique component in *Dendrobium nobile* Lindl. is thought to be due to their higher content compared to other *Dendrobium nobile* species. Jin et al. (1981) compared the alkaloid content of 11 dendrobium species and found that the alkaloid content of *Dendrobium nobile* Lindl. was much higher than that of other species. The alkaloid content was found to be related not only to the dendrobium variety but also to the age of growth and position of distribution. At the same time, one study found that the content of dendrobium in different clusters and different year branches of *Dendrobium nobile* Lindl. was different, and that the year of growth was the main factor affecting the content of dendrobium in different branches of the same cluster, first year > second year > third year. Lu et al. (2020) and Yan et al. (2018) collected the first-, second-, and third-year *Dendrobium nobile* Lindl. in Sichuan province and determined the content of dendrobium and total alkaloids in their stems. The results showed that the total alkaloid and dendrobium alkaloid contents in the stems of *Dendrobium nobile* Lindl. were first year > second year > third year. The average annual alkaloid content in the upper and lower parts of the stem was 0.77 mg/g and 0.51 mg/g, respectively, indicating that the alkaloid content in the upper part of the stem was higher than the lower part of the stem.

**TABLE 2:** Alkaloids isolated from *Dendrobium nobile* Lindl.

Name of the compound	Molecular formula	Number in Figure 1
Dendrobine	C_16_H_25_NO_2_	1
Nobilonine	C_17_H_27_NO_3_	2
Dendroxine	C_17_H_25_NO_3_	3
Dendrine	C_19_H_29_NO_4_	4
6-Hydroxydendroxine	C_15_H_22_NO_4_	5
8-Hydroxydendroxine	C_15_H_23_NO_4_	6
Nobilomethylene	C_15_H_20_O_3_	7
3-Hydroxy-2-oxodendrobine	C_16_H_25_NO_4_	8
Nordendrobine	C_16_H_26_NO_2_	9
Mubironine B	C_15_H_23_NO_2_	10
Dendroxine	C_17_H_25_NO_3_	11
Dendronobiline A	C_19_H_29_NO_3_	12
Adenosine	C_10_H_13_N_5_O_4_	13
9-Hydroxy-10-oxodendrobine	C_16_H_23_NO_4_	14
N-isopentenyl-6-hydroxydendroxinium	C_22_H_34_NO_4_	15
N-isopentenyldendro xinuum	C_22_H_34_NO_3_	16
N-trans-cinnamoyl tyramine	C_17_H_17_NO_2_	17
N-trans-feruloyl tyramine	C_18_H_19_NO_4_	18

### 2.2 Polysaccharides

**FIGURE 2:**
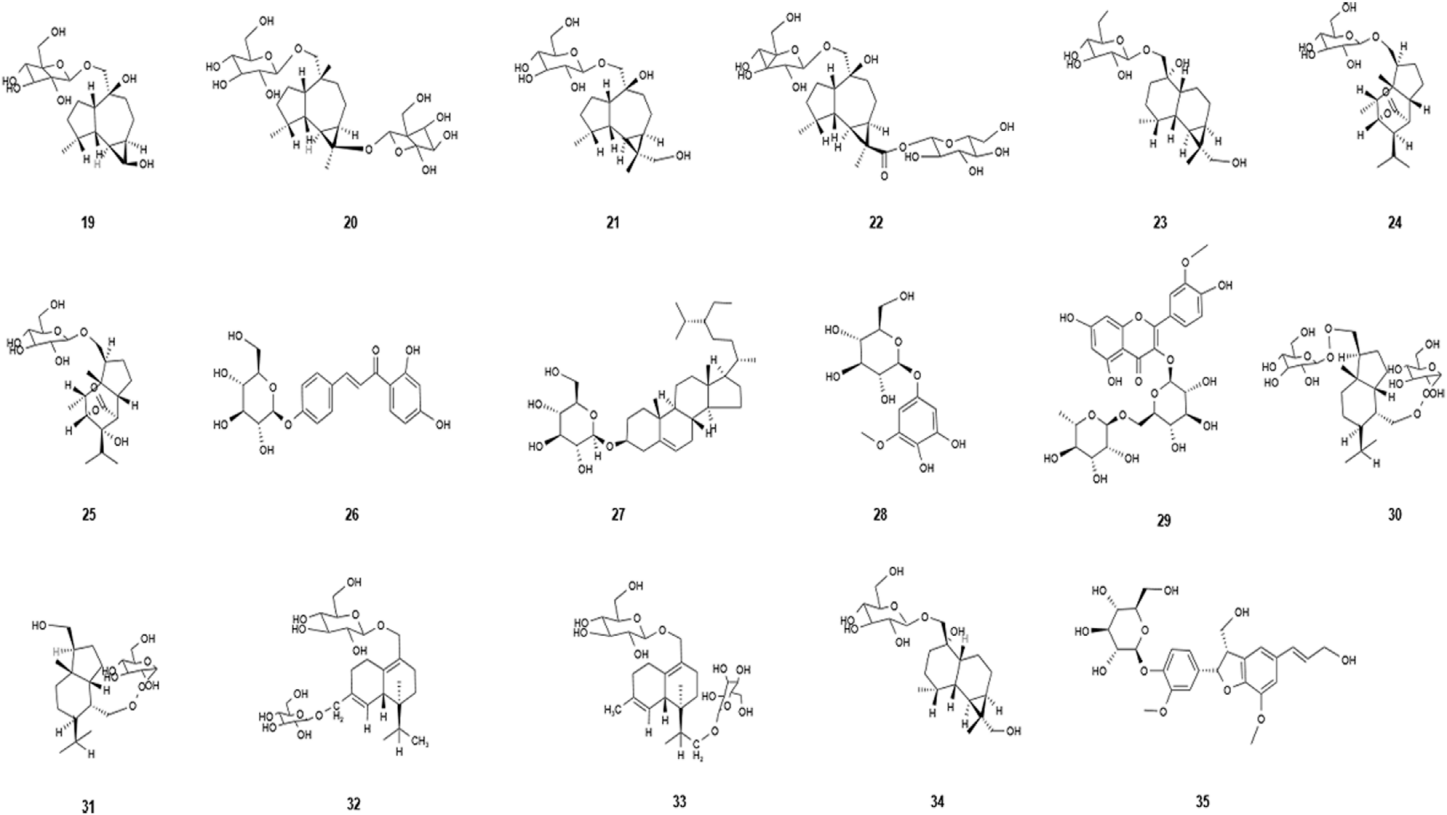
Diagram of the chemical structure of polysaccharides isolated from *Dendrobium nobile* Lindl. Different numbers represent the different compounds: 19, dendroside A; 20, dendroside B; 21, dendroside C; 22, dendroside D; 23, dendroside E; 24, dendroside F; 25, dendroside G; 26, isoliquiritin; 27, daucosterol; 28, koaburaside; 29, isorhamnetin-3-O-β-D-rutinoside; 30, dendronobiloside A; 31, dendronobiloside B; 32, dendronobiloside C; 33, dendronobiloside D; 34, dendronobiloside E; and 35, dehydrodiconiferyl-alcohol-4-β-D-glucoside.

Polysaccharides are biopolymers composed of more than 10 kinds of monosaccharides and are one of the active ingredients in traditional Chinese medicines (TCMs) (Chen et al., 2016). Polysaccharides of *Dendrobium nobile* Lindl. are one of the main active pharmacological ingredients of *Dendrobium nobile* Lindl. The polysaccharides are mainly obtained by extracting and isolating four parts of *Dendrobium nobile* Lindl.: stems, leaves, flowers, and roots, which have a positive effect on anti-tumor, promotion of immune response, anti-oxidation, and anti-inflammation. The different chemical structures of polysaccharides are of great significance for their pharmacological activity. The polysaccharides of *Dendrobium nobile* Lindl. are often composed of several components with different molecular weight ranges and different monosaccharides with different molecular weights. The polysaccharides of *Dendrobium nobile* Lindl. consist mainly of mannose, glucose, and galactose, with rhamnose, rham arabinose, and xylose in small amounts (Jiang and Luo, 2011). Several types of polysaccharides have been identified in *Dendrobium nobile* Lindl. (Wang et al., 2017; Zhang et al., 2016b; Pan et al., 2014). The names and molecular formula of the polysaccharides isolated from *Dendrobium nobile* Lindl. are listed in Table 3, and their chemical structures are shown in Figure 2. Polysaccharides with different compositions are composed of different monosaccharides and have different biological activities. The polysaccharide content of *Dendrobium nobile* Lindl. also varies depending on the site and growth period. Zhang et al. (2013) found that the polysaccharide content of *Dendrobium nobile* Lindl. varies depending on the site, habitat, and growth period. Based on the polysaccharide content in different organs, the polysaccharide content of the two dendrobium species showed a trend of stem > leaf > flower > root, with a significant difference between them. Similarly, in one study, the accumulation of water-soluble polysaccharides in *Dendrobium nobile* Lindl. first increased with prolonged growth period and then decreased and saturated in the first year, reaching the highest value of 2.83% in the second year, and the water-soluble polysaccharide content decreased to 2.25% in the third year (Yang et al., 2010).

**TABLE 3:** Polysaccharides isolated from *Dendrobium nobile* Lindl.

Name of the compound	Molecular formula	Number in Figure 2
Dendroside A	C_27_H_48_O_12_	19
Dendroside B	C_21_H_38_O_8_	20
Dendroside C	C_29_H_40_O_8_	21
Dendroside D	C_27_H_44_O_14_	22
Dendroside E	C_21_H_36_O_8_	23
Dendroside F	C_21_H_34_O_9_	24
Dendroside G	C_21_H_34_O_10_	25
Isoliquiritin	C_21_H_22_O_9_	26
Daucosterol	C_35_H_60_O_6_	27
Koaburaside	C_14_H_20_O_9_	28
Isorhamnetin-3-O-β-D-rutinoside	C_28_H_32_O_16_	29
Dendronobiloside A	C_27_H_48_O_12_	30
Dendronobiloside B	C_21_H_38_O_8_	31
Dendronobiloside C	C_27_H_44_O_12_	32
Dendronobiloside D	C_27_H_44_O_12_	33
Dendronobiloside E	C_28_H_45_O_12_	34
Dehydrodiconiferyl-alcohol-4-β-D-glucoside	C_26_H_32_O_11_	35

### 2.3 Bibenzyls

**FIGURE 3:**
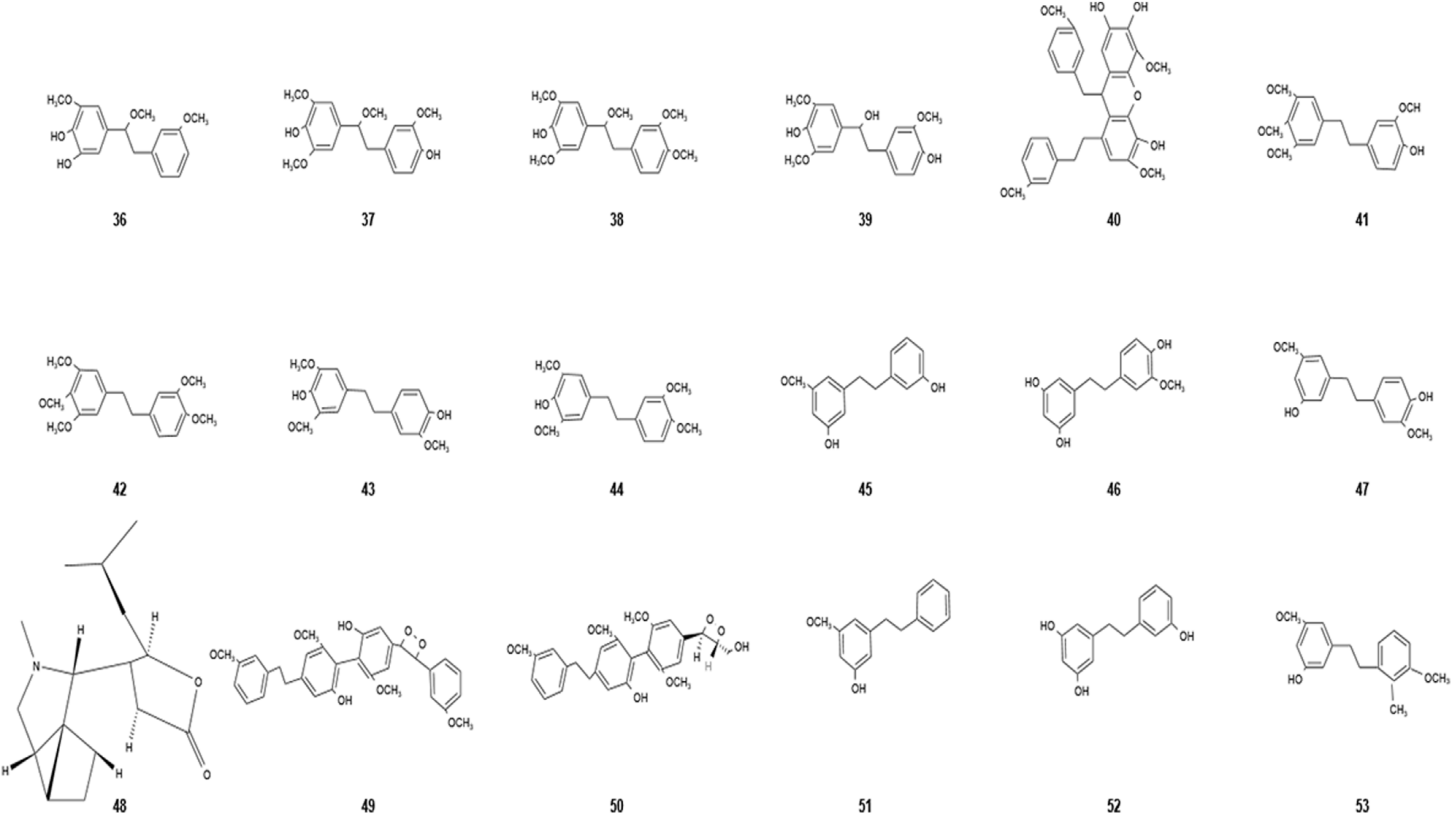
Diagram of the chemical structure of bibenzyls isolated from *Dendrobium nobile* Lindl. Different numbers represent the different compounds: 36, nobilin A; 37, nobilin B; 38, nobilin C; 39, nobilin D; 40, nobilin E; 41, crepidatin; 42, chrysotobibenzyl; 43, moscatilin; 44. chrysotoxine; 45, batatasin III; 46, tristin; 47, gigantol; 48, (-)-dendrobin; 49, dendronophenol A; 50, dendronophenol B; 51, 3-hydroxy-5-methoxybibenzyl; 52, 3,3′,5-trihydroxybibenzyl; and 53, 3-O-methylgigantol.

Bibenzyls are a class of compounds consisting of two benzyl units attached to a methyl group by a single C–C bond. They are found in a variety of plants, including mosses, ferns, and angiosperms. Dendrobium, a type of orchid, is a common medicinal plant for bibenzylate extraction. It is also a type of active compound that is abundantly contained in dendrobium, an herbal medicine. Bibenzyls are mainly extracted from the stems of *Dendrobium nobile* Lindl. (Xiao et al., 2016). It has attracted much attention in recent years due to its excellent anti-tumor activity (Kou et al., 2013). Luo et al. (2006) isolated 31 monomeric compounds from *Dendrobium nobile* Lindl. by the thiazolyl blue method. The results showed that three benzenes, namely, crepidatin, chrysotobibenzyl, and moscatilin, exhibit a certain growth inhibitory effect against highly invasive human hepatocellular carcinoma cell lines. With the development of extraction technology, researchers have continuously discovered new bibenzyls from *Dendrobium nobile* Lindl. (Chao et al., 2018). The basic information of bibenzyls from *Dendrobium nobile* Lindl. has been shown in Table 4 and Figure 3. The bibenzyls also have good anti-oxidant activity. Zhang et al. (2008b) isolated and identified the chemical components in a 60% ethanol aqueous extract of *Dendrobium nobile* Lindl. and obtained three new bibenzyl compounds, namely, nobilin A, nobilin B, and nobilin C. The activity of these compounds was confirmed *in vitro* by 1, 1-diphenyl-2-picrylhydrazyl (DPPH) radical scavenging and oxygen-free radical scavenging methods.

**TABLE 4:** Bibenzyls isolated from *Dendrobium nobile* Lindl.

Name of the compound	Molecular formula	Number in Figure 3
Nobilin A	C_17_H2_0_O_5_	36
Nobilin B	C_18_H_23_O_6_	37
Nobilin C	C_19_H_25_O_6_	38
Nobilin D	C_17_H2_0_O_6_	39
Nobilin E	C_32_H_32_O_8_	40
Crepidatin	C_18_H_22_O_5_	41
Chrysotobibenzy	C_19_H_24_O_5_	42
Moscatilin	C_21_H_24_O_7_	43
Chrysotoxine	C_18_H_22_O_5_	44
Batatasin III	C_15_H_16_O_3_	45
Tristin	C_15_H_16_O_4_	46
Gigantol	C_16_H_18_O_4_	47
(-)-Dendrobin	C_16_H_25_NO_2_	48
Dendronophenol A	C_32_H_32_O_8_	49
Dendronophenol B	C_27_H_30_O_8_	50
3-Hydroxy-5-methoxybibenzyl	C_15_H_16_O_2_	51
3,3′,5-Trihydroxybibenzyl	C_14_H_14_O_3_	52
3-*O*-methylgigantol	C_17_H_20_O_4_	53

### 2.4 Phenanthrenes

**FIGURE 4:**
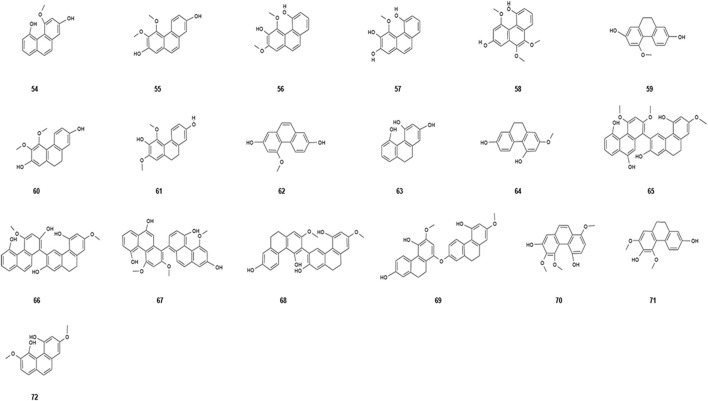
Diagram of the chemical structure of phenanthrenes isolated from *Dendrobium nobile* Lindl. Different numbers represent the different compounds: 54, moscatin; 55, nudol; 56, bulbophyllanthrin; 57, fimbriol B; 58, plicatol A; 59, coelonin; 60, erianthridin; 61, flavanthridin; 62, flavanthrinin; 63, hircinol; 64, lusianthrin; 65, denthyrsinol A; 66, denthyrsinol B; 67, denthyrsinol C; 68, phochinenin G; 69, phochinenin D; 70, 3,4,8-trimethoxyphenanthrene-2,5-diol; 71, 5,7-dimethoxyphenanthrene-2,6-diol; and 72, cannithrene-2.

Phenanthrene has a wide range of biological activities, but has only been reported in a few families in the plant kingdom, and orchids are the most crucial source of natural phenanthrene (Ling et al., 2021). Phenanthrene is a polycyclic aromatic hydrocarbon composed of three benzene rings*. Dendrobium nobile* Lindl. is a plant of the orchid family, and phenanthrene is one of the main components of *Dendrobium nobile* Lindl. and is a substance that has been the subject of much research attention. Subsequently, phenanthrene compounds were continuously extracted and separated from the *Dendrobium nobile* Lindl. (Zhang et al., 2019; Li et al., 2023). The phenanthrene compounds isolated from *Dendrobium nobile* Lindl. are listed in Table 5, and their chemical structure information are shown in Figure 4. Phenanthrene compounds are important compounds for studying the anti-tumor effects of *Dendrobium nobile* Lindl., and several phenanthrene compounds have been found to possess varying degrees of anti-tumor activity. Phenanthrenes are mainly extracted from the dried stems of *Dendrobium nobile* Lindl. (Ling et al., 2021; Zhou et al., 2018b). Zhou et al. (2018b) extracted, isolated, and purified the natural products from the extract of *Dendrobium nobile* Lindl. with different chromatographic techniques to isolate four phenanthrene compounds, namely, densiflorolB, cypripedin, moscatin, and 2meme 4-trimethoxy-phenanthrene-3-diol, and were investigated in human breast cancer MCF-7 cells. The results showed that the phenanthrene compounds had a significant inhibitory effect on breast cancer cells, providing a strong basis for anti-tumor studies of phenanthrene compounds in *Dendrobium nobile* Lindl.

**TABLE 5:** Phenanthrenes isolated from *Dendrobium nobile* Lindl.

Name of the compound	Molecular formula	Number in Figure 4
Moscatin	C_15_H_12_O_3_	54
Nudol	C_16_H_14_O_4_	55
Bulbophyllanthrin	C_16_H_14_O_4_	56
Fimbriol B	C_15_H_12_O_4_	57
Plicatol A	C_17_H_16_O_5_	58
Coelonin	C_15_H_14_O_3_	59
Erianthridin	C_16_H_16_O_4_	60
Flavanthridin	C_16_H_16_O_4_	61
Flavanthrinin	C_15_H_12_O_3_	62
Hircinol	C_30_H_22_O_6_	63
Lusianthrin	C_30_H_26_O_6_	64
Denthyrsinol A	C_30_H_26_O_6_	65
Denthyrsinol B	C_30_H_20_O_5_	66
Denthyrsinol C	C_30_H_22_O_6_	67
Phochinenin G	C30H26O6	68
Phochinenin D	C_30_H_26_O_6_	69
3,4,8-Trimethoxyphenanthrene-2,5-diol	C_17_H_14_O_5_	70
5,7-Dimethoxyphenanthrene-2,6-diol	C_16_H_14_O_4_	71
Cannithrene-2	C_16_H_16_O_4_	72

### 2.5 Sesquiterpenes

**FIGURE 5:**
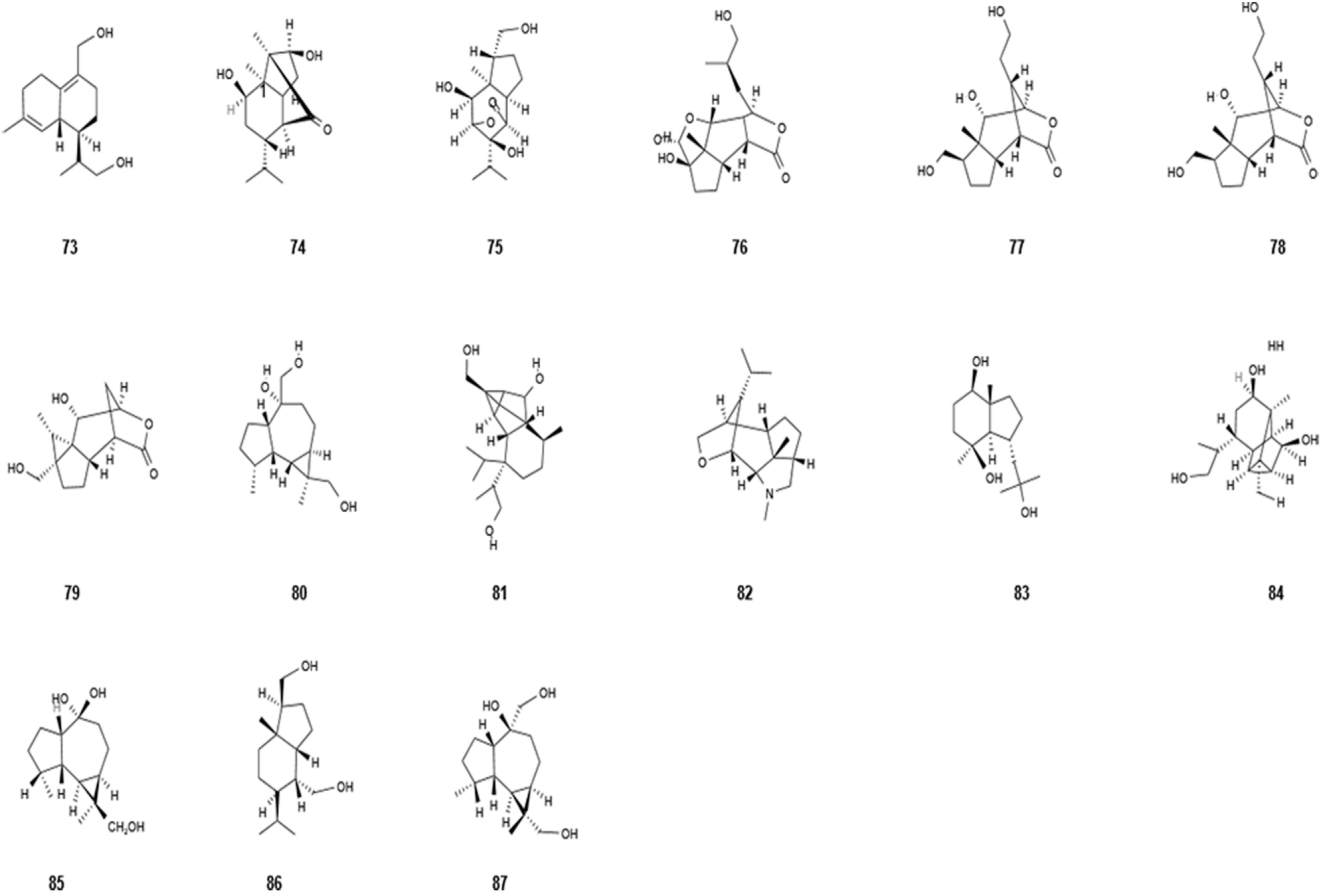
Diagram of the chemical structure of sesquiterpenes isolated from *Dendrobium nobile* Lindl. Different numbers represent the different compounds: 73, δ-cadinen-12,14-diol; 74, dendronobilin A; 75, dendronobilin B; 76, dendronobilin C; 77, dendronobilin D; 78, dendronobilin E; 79, dendronobilin F; 80, dendronobilin H; 81, dendronobilin I; 82, dendrobane A; 83, bullatantirol; 84, dendrobiumane A; 85, 6α,10,12-trihydroxypicrotoxane; 86, 10,12-dihydroxypicmtoxane; and 87, 10β,13,14-trihydroxyalloaromadendrane.

*Dendrobium nobile* Lindl. contains numerous sesquiterpenes, including picrotoxane, isovanillin, cyclocopacamphane and copacamphane, and juniperane sesquiterpenes. In addition, 10 sesquiterpenes have also been extracted and discovered (Wang P. et al., 2019), which has promoted the investigation and application of sesquiterpenes. In the literature, sesquiterpenes in *Dendrobium nobile* Lindl. have been found to have neuroprotective, immunomodulatory, diabetic, anti-tumor, and ameliorating effects on acute cerebral ischemia. Zhang et al. (2007) isolated eight sesquiterpenoids from ethanol–water extract of its dried stems and identified one of them as a new compound, dendronobilin J. The compound dendrodensiflorol was isolated from *Dendrobium nobile* for the first time, and the compound bullatantirol was isolated from *Dendrobium* for the first time. Based on the remarkable biological activity of sesquiterpenes, the sesquiterpenes of *Dendrobium nobile* Lindl. have attracted attention of scholars. The detailed information of sesquiterpene compounds has been shown in Table 6 and Figure 5.

**TABLE 6:** Sesquiterpenes isolated from *Dendrobium nobile* Lindl.

Name of the compound	Molecular formula	Number in Figure 5
δ-Cadinen-12,14-diol	C_15_H_24_O_2_	73
Dendronobilin A	C_15_H_24_O_3_	74
Dendronobilin B	C_15_H_24_O_5_	75
Dendronobilin C	C_15_H_22_O_6_	76
Dendronobilin D	C_15_H_24_O_5_	77
Dendronobilin E	C15H24O5	78
Dendronobilin F	C_15_H_22_O_5_	79
Dendronobilin H	C_15_H_26_O_3_	80
Dendronobilin I	C_18_H_30_O_3_	81
Dendrobane A	C_16_H_27_NO	82
Bullatantirol	C_15_H_28_O_3_	83
Dendrobiumane A	C_15_H_24_O_3_	84
6α,10,12-Trihydroxypicrotoxane	C_15_H_28_O_3_	85
10,12-Dihydroxypicmtoxane	C_15_H_28_O_2_	86
10β,13,14-Trihydroxyalloaromadendrane	C_15_H_26_O_3_	87

### 2.6 Tannins

Modern pharmacological studies show that tannins have a variety of biological activities, such as bacteriostasis, anti-virus, anti-oxidation, anti-tumor, and inhibition of gastrointestinal motility, and are widely used in the fields of food and medicine. The extractions of tannins are currently obtained by the isolation and purification of *Dendrobium nobile* Lindl. stems. Chen et al. determined and compared the content of tannin in *Dendrobium nobile* Lindl., *Dendrobium denneanum* Kerr, *Dendrobium chrysotoxum* Lindl., and *Dendrobium fimbriatum* Hook., and the results showed that *Dendrobium nobile* Lindl. had the highest content, and the contents of *Dendrobium denneanum* Kerr and *Dendrobium fimbriatum* Hook. were similar (Hen et al., 2013). However, there are few studies on the effects of *Dendrobium nobile* Lindl. tannin compounds on diseases, which need further scientific exploration.

### 2.7 Other chemical compositions

**FIGURE 6:**
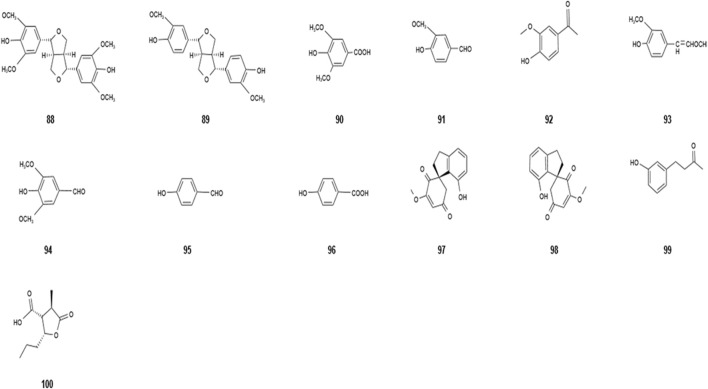
Diagram of the chemical structure of other chemical compositions isolated from *Dendrobium nobile* Lindl. Different numbers represent the different compounds: 88, syringaresinol; 89, pinoresinol; 90, syringic acid; 91, vanillin; 92, apocynin; 93, coniferyl aldehyde; 94, syringaldehyde; 95, p-hydroxybenzaldehyde; 96, p-hydroxybenzoic acid; 97, (+)-denobilone A; 98, (-)-denobilone A; 99, 4-(3-hydroxyphenyl)-2-butanone; and 100, deeumbic acid A.

Through research and chemical extraction by scientific researchers, it was found that *Dendrobium nobile* Lindl. has other components such as fluorenone, phenolic acid, coumarin, and lignans in addition to the aforementioned chemical composition (Zhou et al., 2018a; Wang, 2021). The detailed information of the other chemical compositions has been shown in Table 7 and Figure 6. *Dendrobium nobile* Lindl. has high medicinal value as a valuable herbal medicine, and it would be beneficial to better understand, develop, and utilize *Dendrobium nobile* Lindl. by extracting the different chemical constituents.

**TABLE 7:** Other chemical compositions isolated from *Dendrobium nobile* Lindl.

Name of the compound	Molecular formula	Number in Figure 6
Syringaresinol	C_22_H_26_O_8_	88
Pinoresinol	C_20_H_22_O_6_	89
Syringic acid	C_9_H_10_O_5_	90
Vanillin	C_8_H_8_O_3_	91
Apocynin	C_24_H_20_O_10_	92
Coniferyl aldehyde	C_10_H_10_O_3_	93
Syringaldehyde	C_9_H_10_O_4_	94
*p*-hydroxybenzaldehyde	C_7_H_6_O_2_	95
*p*-hydroxybenzoic acid	C_7_H_6_O_3_	96
(+)-Denobilone A	C_15_H_14_O_4_	97
(-)-Denobilone A	C_15_H_14_O_4_	98
4-(3-Hydroxyphenyl)-2-butanone	C_10_H_12_O_2_	99
Deeumbic acid A	C_12_H_14_O_4_	100

In summary, the study of the chemical composition of *Dendrobium nobile* Lindl. focused mainly on alkaloids and polysaccharide compounds because of the realistically high alkaloid and polysaccharide content of *Dendrobium nobile* Lindl. and because the study was conducted earlier. Then, they were studied from many aspects, including their corresponding pharmacological activity and the effects of different cultivation conditions and different plant parts on their content. At present time, most of the chemical active ingredients found in *Dendrobium nobile* Lindl. are extracted from the stem part of *Dendrobium nobile* Lindl., such as alkaloids, polysaccharides, bibenzylates, and phenanthrenes. In addition to the stem, alkaloids and polysaccharides can also be extracted from the leaves of *Dendrobium nobile* Lindl., and polysaccharide compounds can also be extracted from the flowers and roots of *Dendrobium nobile* Lindl. With the development of new extraction techniques and detailed studies of the pharmacological activity of *Dendrobium nobile* Lindl., the discovery of bibenzyls, phenanthrenes, sesquiterpenes, and coumarins gradually attracted people’s attention and became a new direction for the pharmacological activity research of *Dendrobium nobile* Lindl. For example, studies have shown that bibenzyls and phenanthrenes have certain inhibitory effects on certain types of tumor cells. At present, however, studies on the chemical constituents of *Dendrobium nobile* Lindl. are mainly alkaloids and polysaccharides, with few reports on other chemical constituents.

## 3 Experimental research progress of *Dendrobium nobile* Lindl. in diseases

*Dendrobium nobile* Lindl., a well-known herbal medicine, has become a research hotspot due to its abundant pharmacological activity and high medicinal value. This has led researchers to conduct many experimental studies on the therapeutic mechanisms of *Dendrobium nobile* Lindl. in various diseases in order to better understand its pharmacological actions and therapeutic mechanisms. Currently, experimental research on *Dendrobium nobile* Lindl. is focused primarily on the oncologic, nervous system, cardiovascular, ophthalmic, and gastrointestinal diseases.

### 3.1 *Dendrobium nobile* Lindl. and tumor diseases

Many studies have demonstrated that *Dendrobium nobile* Lindl. has a marked inhibitory effect on a variety of tumor cells (Zhang et al., 2021). In the conventional view, phenanthrenes and bibenzyls were considered to be the major anti-tumor compounds of *Dendrobium nobile* Lindl. Three bibenzyl components of *Dendrobium nobile* Lindl.: crepidatin, chrysotobibenzyl, and moscatilin showed different growth inhibitory effects against human hepatocellular carcinoma cell line FHCC-98, and among them, the effect of moscatilin was particularly evident (Luo et al., 2006). Three phenanthrene compounds isolated from *Dendrobium nobile* Lindl. were also found to significantly inhibit the growth of human breast cancer MCF-7 cells (Zhou et al., 2018b). Nudol, a phenanthrene compound derived from *Dendrobium nobile* Lindl., was reported to arrest the U2OS cell cycle in the G2/M phase and induce apoptosis in a caspase-dependent manner. In addition, treatment with Nudol inhibited U2OS cell migration (Zhang et al., 2019). With a deeper understanding of the active components of *Dendrobium nobile* Lindl., other components of *Dendrobium nobile* Lindl., such as polysaccharides and alkaloids, have also been found to have anti-tumor effects. Polysaccharides of *Dendrobium nobile* Lindl. have been reported to suppress the BCR-ABL fusion gene mRNA expression in K562 cells, directly inhibit proliferation, and induce apoptosis of chronic myeloid leukemia K562 cells (Zheng, 2010). Ge et al. (2015) found that polysaccharides of *Dendrobium nobile* Lindl. have a killing effect on leukemia cells and elucidated the mechanism of this effect. He et al. (2017) also observed the effects of a fat-soluble alkaloid extract of *Dendrobium nobile* Lindl. on HT-29 colon cancer cells by *in vitro* cell experiments and found that the fat-soluble alkaloid extract of *Dendrobium nobile* Lindl. reduced the survival rate of HT-29 colon cancer cells and 29 cells, induces apoptosis of HT-29 cells, and inhibits the G2 phase of the cell cycle, thereby inhibiting colon cancer cell growth. The mechanism may be due to the liposoluble alkaloid extract of *Dendrobium nobile* Lindl. decreasing mitochondrial membrane potential, increasing intracellular reactive oxygen species concentration, and increasing the expression of activated caspases 9 and 3 and intracellular cytochrome.

### 3.2 *Dendrobium nobile* Lindl. and nervous system diseases

Recent studies have confirmed that alkaloids, one of the active components of *Dendrobium nobile* Lindl., have a significant protective effect on the nervous system and play a certain role in treatment. Studies have shown that *Dendrobium nobile* Lindl. alkaloid (DNLA), the active ingredient of the Chinese herb *Dendrobium*, reduces the cytotoxicity of amyloid-β protein fragments 25–35 (Aβ25–35) on primary cultured rat neurons and protect against synaptic integrity in cultured neurons by DNLA. The mechanism may be mediated, at least in part, through upregulation of the neurogenesis-related proteins synaptophysin and postsynaptic density-95 (Zhang et al., 2017). Li et al. (2017) found that as a pathological signal of early Alzheimer’s disease, total alkaloids of *Dendrobium nobile* Lindl. increase autophagic flow by promoting the formation and degradation of autophagosomes in the hippocampus, thereby increasing Aβ25-35 induced axonal degeneration and preventing the development of Alzheimer’s disease (AD). Another study on the effect of DNLA on a rat model of Aβ25-35-induced dementia and its mechanism found that dendrobium alkaloids can improve the content of β-amyloid (Aβ1-42), amyloid precursor protein (APP), and β-site APP cleaving enzyme (BACEI) proteins in the hippocampus, providing a valid basis for the use of DNLA in AD treatment (Zhang et al., 2016a). In addition to improving AD, *Dendrobium nobile* Lindl. also has the ability to improve memory. For example, the *Dendrobium nobile* Lindl. alkaloid significantly ameliorates damage and loss of hippocampal neurons by streptozotocin in rats, while simultaneously activating glycogen-synthase kinase-3β (GSK-3b) and inhibiting tau hyperphosphorylation (Ba et al., 2017). Wang et al. (2016a) found that *Dendrobium nobile* Lindl. polysaccharide attenuated lipopolysaccharide-induced learning memory impairment and neuronal damage in rats. They found that *Dendrobium nobile* Lindl. polysaccharide could attenuate learning memory impairment and neuronal damage caused by lipopolysaccharides in rats and inhibit inflammation of the hippocampus. The mechanism was to improve learning and memory and to reduce gene and protein expression of tumor necrosis factor-α (TNF-α), interleukin-1β (IL-1β), and transforming growth factor-β1 (TGF-β1) in anti-lipopolysaccharide-induced rat hippocampal neurons. In addition to the prevention of AD and improving memory function, several components of *Dendrobium nobile* Lindl. can protect neurons and nerve cells. Among them, the polysaccharides of *Dendrobium nobile* Lindl. protect neurons by inhibiting the activation of lipopolysaccharides in microglia and astrocytes and reducing the production of inflammatory cytokines (Lin et al., 2016). Thus, *Dendrobium nobile* Lindl. has a great potential for the treatment and protection of nervous system diseases.

### 3.3 *Dendrobium nobile* Lindl. and cardiovascular diseases

Hyperlipidemia causes not only vascular damage and atherosclerosis-related complications due to atherosclerosis but also forms atherosclerotic plaques in large and medium arteries, which develop and damage the vessel wall, causing numerous dangerous lesions in the vessel wall that are a major cause of cardiovascular disease. Hyperglycemia can affect blood vessels and predispose to cardiovascular disease and myocardial infarction; *Dendrobium nobile* Lindl. has favorable hypoglycemic and lipid-lowering effects and is expected to contribute to the prevention and treatment of cardiovascular disease. Polysaccharides of *Dendrobium nobile* Lindl. have been reported to reduce total cholesterol (TC), triacylglycerol (TG), and low-density lipoprotein cholesterol (LDL-C) in the serum of hyperlipemic rats. In hyperlipidemic rats, it increases high-density lipoprotein cholesterol (HDL-C), decreases liver index and malondialdehyde (MDA) levels in liver tissue, and increases superoxide dismutase (SOD) activity to reduce hepatic adiposity (Li et al., 2008). In addition, total alkaloids of *Dendrobium nobile* Lindl. were found to attenuate insulin resistance and contribute to hypoglycemia by modulating IRS-2 mRNA and IGF-1 mRNA levels in hepatocytes (Huang et al., 2014). Furthermore, aqueous extracts of *Dendrobium nobile* Lindl. have the potential to treat hyperlipidemia by lowering serum triglyceride and low-density lipoprotein cholesterol concentrations, decreasing alanine aminotransferase (ALT) activity, and increasing high-density lipoprotein cholesterol concentrations, and aqueous extracts of *Dendrobium nobile* Lindl. solution have significant lipid-lowering activity, providing experimental evidence for the prevention and treatment of cardiac diseases (Li et al., 2019).

### 3.4 *Dendrobium nobile* Lindl. and ophthalmic diseases

There are a number of herbal medicines developed from *Dendrobium nobile* Lindl. for the treatment of ophthalmic diseases. A thorough study of the pharmacological effects of *Dendrobium nobile* Lindl. found that *Dendrobium nobile* Lindl. has a better therapeutic effect on cataracts based on its anti-oxidant, anti-inflammatory, glucose-lowering, and protective effects on oxidized epithelial cells. Recent studies have shown that alkaloids of *Dendrobium nobile* Lindl. can affect nitric oxide (NO) and inducible nitric oxide synthase (iNOS) activity and their gene expression in the lens of galactose diabetic cataract rats, effectively suppressing the iNOS gene expression, which is diabetic with a better therapeutic effect on cataracts (Wei and Long, 2008). Another study showed that the total alkaloids and crude polysaccharides of *Dendrobium nobile* Lindl. have certain anti-cataract effects *in vitro*, the mechanism is related to their antagonistic effects on oxidative damage in the lens, and the total alkaloids of *Dendrobium nobile* Lindl. have been demonstrated to have a superior anti-cataract effect than polysaccharides (Long et al., 2008). In addition to cataracts, another high-incidence ophthalmic disease, diabetic retinopathy (DR) also damages the human visual system. Considering the anti-inflammatory, anti-oxidant, hypoglycemic, angiogenesis-inhibiting, and retinal damage caused by hyperglycemia, it is clear that reactive oxygen species and inflammatory substances are major factors in DR. Therefore, the use of active ingredients in dendrobium for the treatment of DR has become a hot topic, but there is little literature on the treatment of DR with *Dendrobium nobile* Lindl. The role of other forms of dendrobium in the treatment of DR, such as *Dendrobium chrysotoxum* Lindl. and *Dendrobium officinale*, have also been studied. Yu et al. (2016) observed the ameliorative effect of an ethanol extract of *Dendrobium chrysotoxum* Lindl. on mice with diabetic retinopathy and found that *Dendrobium chrysotoxum* Lindl. induced a significant reduction of hypoxia inducible factor-1α (HIF-1α) and vascular endothelial growth factor (VEGF) and its receptors VEGFRl and VEGFR2 in the retina of DR mice, and that it could reduce the elevated levels of VEGF in the vitreous and serum of DR mice. Furthermore, it suppresses retinal neovascularization in DR. Furthermore, *Dendrobium officinale* polysaccharide reduced retinal and systemic inflammatory cytokine (IL-6 and TNF-α) levels and inhibited the upregulation of retinal VEGF expression in DR rats (Li et al., 2016).Currently, there is no literature on the prophylactic and therapeutic effects of *Dendrobium nobile* Lindl. on DR. Thus, *Dendrobium nobile* Lindl. has a large research space in the prevention and treatment of DR.

### 3.5 *Dendrobium nobile* Lindl. and gastrointestinal diseases

A recent study showed that *Dendrobium nobile* Lindl. was effective in increasing ileogastric levels, decreasing growth inhibitors, increasing the rate of small intestinal acceleration, and improving constipation symptoms in a rat model (Gan et al., 2019). Thus, *Dendrobium nobile* Lindl. may be of clinical value in the treatment of gastrointestinal disorders. In addition, *Dendrobium nobile* Lindl. extract could increase stool water content, promote intestinal peristalsis, regulate intestinal lactate, and improve symptoms of constipation in mice through a mouse model (Wang et al., 2021). Furthermore, *Dendrobium nobile* Lindl. has been reported to promote gastric acid secretion and digestion in humans. Experimentally, *Dendrobium nobile* Lindl. directly stimulates G cells, which is presumed to increase the concentration of gastrin in the blood, resulting in increased gastrin release, higher serum gastrin concentration, and increased acid secretion in the stomach (Chen et al., 1995).

### 3.6 *Dendrobium nobile* Lindl. and other diseases

**FIGURE 7:**
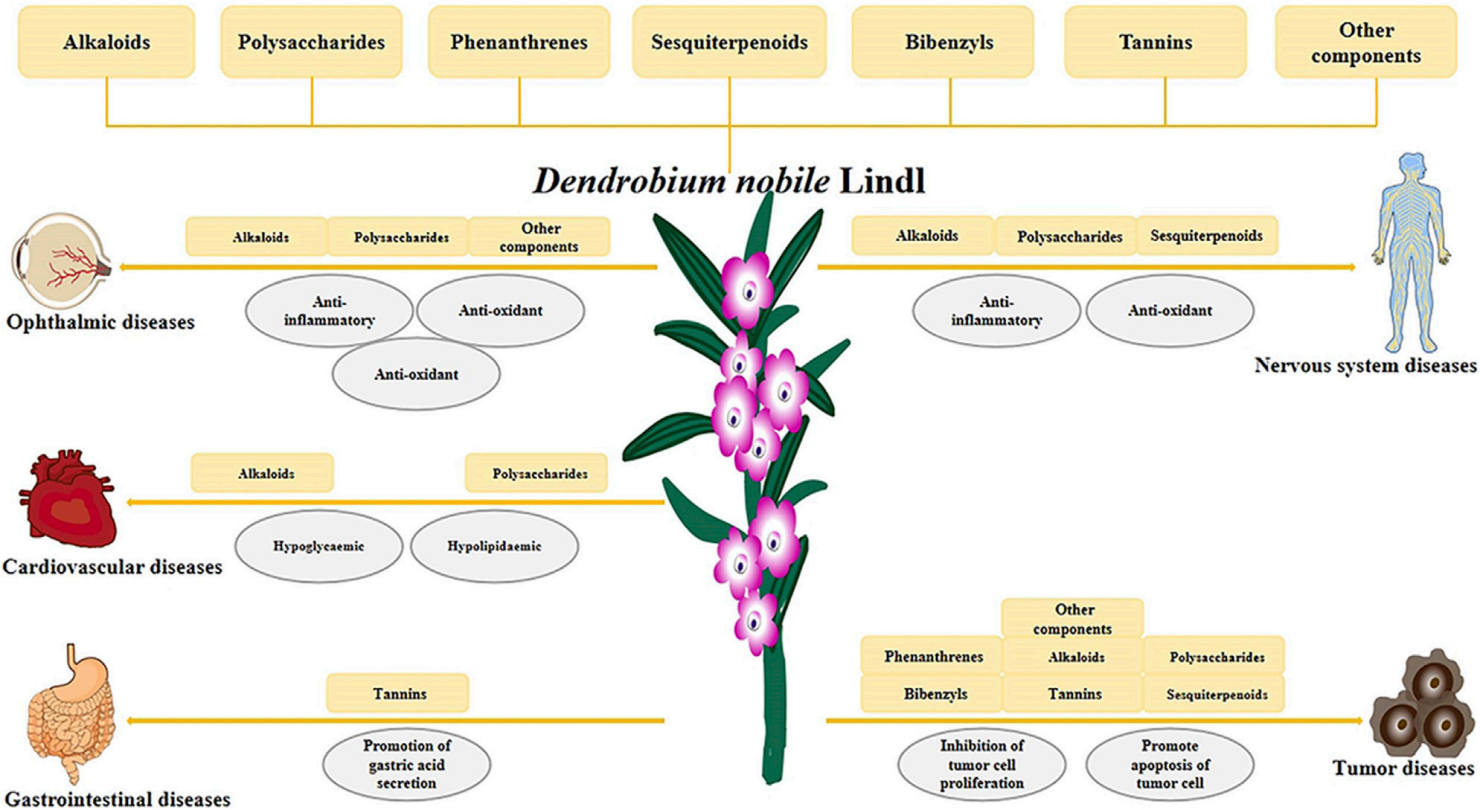
Diagram of the application of *Dendrobium nobile* Lindl. to different diseases. *Dendrobium nobile* Lindl. is composed of alkaloids, polysaccharides, bibenzyls, phenanthrenes, sesquiterpenoids, tannins, and other chemical components, which have good pharmacological activities, such as anti-inflammatory, anti-oxidant and hypoglycemic, hypolipidaemic, inhibition of tumor cell proliferation, promotion of apoptosis of tumor cell, and promotion of gastric acid secretion. Therefore, according to the aforementioned pharmacological activities, the various chemical components of *Dendrobium nobile* Lindl. can effectively target different diseases and play a role in clinical therapeutics.

It has been shown that the immune system is somewhat enhanced by the ingestion of *Dendrobium nobile* Lindl. In animal studies, *Dendrobium nobile* Lindl. powders of different particle sizes were found to significantly increase the spleen index of mice, indicating that *Dendrobium nobile* Lindl. enhances immune function in mice at the organ level. In addition, *Dendrobium nobile* Lindl. also has a protective role in liver function. The alkaloids of *Dendrobium nobile* Lindl. have been reported to improve the hepatic lipid profile of mice through two pathways: promoting cholesterol excretion by facilitating the binding of hydrophilic sulfate to bile acids and reducing the cholic acid/chenodeoxycholic acid (CA/CDCA) ratio, which is positively correlated with cholesterol absorption. This is in line with the findings of the study. This reveals a protective role of *Dendrobium nobile* Lindl. alkaloids in liver lipid homeostasis and possible mechanisms (Huang et al., 2019). Another study showed that the protective effect of *Dendrobium nobile* Lindl. alkaloids against CCl4-induced liver injury was associated with amelioration of mitochondrial oxidative stress and mitochondrial dysfunction, which was dependent on activation of nuclear factor erythroid 2-related factor 2 (Nrf2) signaling (Zhou et al., 2020). In addition, the researchers also found that *Dendrobium nobile* Lindl. had good anti-microbial activity. Zhang et al. (2012) compared the anti-microbial activity of *Dendrobium nobile* Lindl. and *Dendrobium ferruginum*, and the results showed that both types of *Dendrobium* polysaccharides had significant anti-microbial activity on *Staphylococcus aureus*, *Escherichia coli*, and *Pneumococcus*, and the anti-microbial activity of *Dendrobium nobile* Lindl. was more obvious. Another study also found that different *Dendrobium nobile* Lindl. extracts showed good inhibitory effects against five common drug-resistant bacteria *Staphylococcus aureus*, *methicillin-resistant staphylococcus aureus*, *Pseudomonas aeruginosa*, *Escherichia coli*, and *Klebsiella pneumoniae* (Wang et al., 2022). The good anti-microbial activity can not only make *Dendrobium nobile* Lindl. useful in the treatment of infection diseases but also provide a reference for the research and development of new anti-microbial drugs. The aforementioned experimental studies based on the pharmacological effects of the active ingredient of *Dendrobium nobile* Lindl., such as glucose lowering, anti-tumor, anti-oxidant, and anti-inflammatory effects, have shown therapeutic effects on cancer, nervous system, cardiovascular system, ophthalmology, and gastrointestinal diseases (Figure 7), providing a theoretical and experimental basis for further clinical treatment and a scientific reference for the better development of *Dendrobium nobile* Lindl. drugs.

## 4 Summary

*Dendrobium nobile* Lindl. is a common herbal medicine and has a long history of medicinal use. This paper outlines the chemical composition of *Dendrobium nobile* Lindl. and its application in the treatment of clinical diseases. *Dendrobium nobile* Lindl. is increasingly being applied in the treatment of clinical diseases due to its good pharmacological activity and high medicinal value. The main chemical constituents of *Dendrobium nobile* Lindl. are alkaloids, polysaccharides, bibenzyls, phenanthrenes, sesquiterpenes, and tannins. Among these, alkaloids and polysaccharides are the typical bioactive components of *Dendrobium nobile* Lindl. Current research on the chemical composition and pharmacological effects of *Dendrobium nobile* Lindl. is focused on both alkaloids and polysaccharides. In clinical disease treatment studies, *Dendrobium nobile* Lindl. has been shown to have pharmacological effects on cardiovascular disease, neurological disease, anti-tumor, cataract treatment, and digestive disease. More notably, *Dendrobium nobile* Lindl. may play an essential role in the treatment of cardiovascular diseases and diabetes.

At present, there still seems to be some problems in applying the chemical constituents of *Dendrobium nobile* Lindl. to the treatment of clinical diseases. First, numerous studies have focused on crude extracts of *Dendrobium nobile* Lindl., but these crude extracts also contain other chemical constituents, leaving the final results observed largely unexplained. Therefore, more detailed studies are needed to elucidate the pharmacological activity and mechanisms of the chemical constituents in *Dendrobium nobile* Lindl. Second, although the chemical composition and pharmacology of *Dendrobium nobile* Lindl. have been studied to some extent, its pharmacological activity is mainly focused on the treatment of anti-tumor, hypoglycemia, and nervous system diseases, with little reported on its effects on other diseases. Therefore, in order to discuss the efficacy of *Dendrobium nobile* Lindl. against various clinical diseases, it would be necessary to consider the mechanism of the combination of multiple chemical components in addition to the therapeutic effect of a single chemical component on a single disease.
